# Dithioerythritol-capped silver/gold nanoclusters for determination of ciprofloxacin, norfloxacin, and enrofloxacin in food and urine samples†

**DOI:** 10.1039/d5ra02878g

**Published:** 2025-07-07

**Authors:** Mohamed N. Goda, Laila S. Alqarni, Hossieny Ibrahim, Al-Montaser Bellah H. Ali, Mohamed M. El-Wekil

**Affiliations:** a Department of Chemistry, College of Science, Imam Mohammad Ibn Saud Islamic University (IMSIU) Riyadh 11623 Saudi Arabia; b Department of Chemistry, Faculty of Science, Assiut University Assiut 71516 Egypt; c Department of Pharmaceutical Analytical Chemistry, Faculty of Pharmacy, Assiut University Assiut 71516 Egypt mohamed.elwakeel@pharm.aun.edu.eg mohamed.mohamoud@ymail.com

## Abstract

Fluoroquinolones (FQs) are widely used in the poultry and livestock industries due to their effectiveness in preventing and treating bacterial infections. However, improper use and poor biodegradability lead to their accumulation in the food chain, posing risks to human health. To address this, a novel ratiometric fluorescence probe was developed for sensitive FQ detection. The probe consists of dithioerythritol-protected silver–gold nanoclusters (DIT@AgAuNCs) with weak red fluorescence at 615 nm. Addition of Al^3+^ induces nanocluster aggregation, enhancing fluorescence emission. Upon adding FQs to the DIT@AgAuNCs/Al^3+^ system, fluorescence at 615 nm decreases due to the removal of Al^3+^ from the ligand (DIT) surface *via* coordination interactions. Simultaneously, a new blue fluorescence peak emerges at 465 nm, attributed to the formation of an Al^3+^-FQs coordination complex. Under optimal conditions, the fluorescence ratio (F_465_/F_615_) increased proportionally with FQ concentration. In this study, F_465_/F_615_ represents the ratio of fluorescence intensity at 465 nm (blue emission from the Al^3+^-FQ complex) to that at 615 nm (red emission from aggregated DIT@AgAuNCs). This ratiometric approach compensates for environmental and instrumental fluctuations, enhancing analytical reliability. The probe exhibited good linearity for ciprofloxacin (CIP), norfloxacin (NOR), and enrofloxacin (ENR) within the ranges of 0.01–60 μM, 0.018–60 μM, and 0.021–60 μM, respectively, with detection limits 0° 31 nM, 38 nM, and 44 nM. The probe was successfully applied to detect FQs (using CIP as an example) in egg, milk, and urine samples, demonstrating high accuracy with recoveries of 94.0–106.0% and excellent reliability, with an RSD below 4.09%.

## Abstract 2

A DIT@AgAuNCs probe was developed for the fluorometric determination of three structurally related FQs *via* an Al^3+^-induced AIE mechanism.

## Graphical abstract

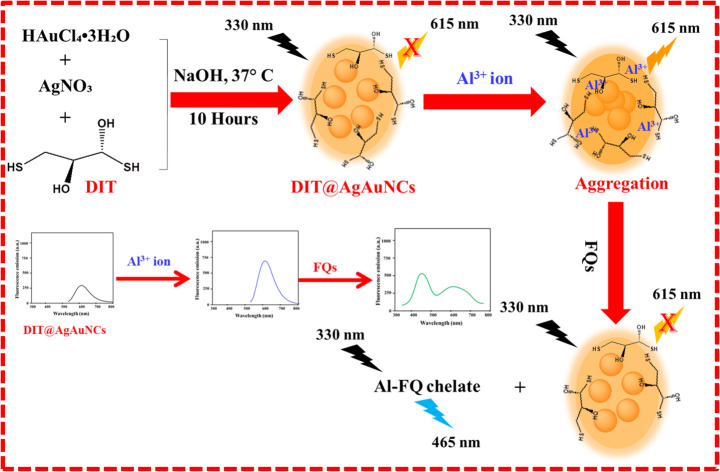

## 1. Introduction

Fluoroquinolones (FQs) are a class of synthetic antibacterial agents extensively used in poultry and livestock.^1^ They are highly stable and resistant to degradation in food chains and environmental waters, leading to their accumulation in the human body and posing a threat to human health.^2^ Traditional methods for detecting FQs include capillary electrophoresis,^3^ liquid chromatography with mass detection,^4^ and liquid chromatography with UV detection.^5^ While these methods offer high selectivity and sensitivity, they are time-consuming, require skilled personnel, and involve extensive sample pretreatment steps.^6–8^ Therefore, developing a simple, rapid, and sensitive method for detecting FQs in various matrices is essential.

Fluorometric-based sensors have gained increased attention in the field of biosensing due to their rapid response, high selectivity, simplicity, and sufficient sensitivity.^9,10^ Fluorometric methods utilizing measurements at two different wavelengths provide a reliable approach for analyte detection, effectively minimizing interferences from external environmental factors, including instrumental and measurement errors.^11,12^ Monometallic nanoclusters (NCs) are ultrasmall nanoparticles, typically composed of a single type of metal atom, with sizes ranging between 1–3 nanometers. These clusters contain only a few to a few hundred atoms, making them smaller than conventional nanoparticles but larger than individual molecules. Their unique electronic, optical, and catalytic properties arise due to quantum size effects and the presence of discrete energy levels, unlike bulk metals that exhibit continuous energy bands.^13^ In contrast, bimetallic nanoclusters offer significant advantages over monometallic NCs, including higher fluorescence quantum yield, larger Stokes shift, and greater stability, making them promising candidates for sensing and imaging applications.^14^ Several studies in the literature have reported the determination of FQ drugs using monometallic nanoclusters. For instance, Hosseini and Sadeghi developed copper nanoclusters (CuNCs) capped with sodium gluconate for the detection of ciprofloxacin (CIP) and ofloxacin (OFL), achieving detection limits of 9.0 nM and 8.0 nM, respectively.^15^ Similarly, Lian *et al.* synthesized cysteine-modified CuNCs for the fluorescence sensing of OFL and norfloxacin (NOR), with a detection limit of 50.0 nM for both.^16^ However, these methods rely on single-emission probes, which are susceptible to interference from external environmental factors. A method to improve the fluorescence emission of NCs is aggregation-induced emission (AIE) effect. Various metals, including aluminum(iii), silver(i), cerium(iii), lanthanum(iii), and zinc(ii), can induce nanocluster (NC) aggregation, enhancing their fluorescence emission. For instance, Zhang *et al.* developed a fluorescence probe based on glutathione (GSH)-capped CuNCs for the detection of *p*-nitrophenol, utilizing cerium(iii)-induced aggregation of GSH-CuNCs.^17^

This study presents a red-emissive DIT@AgAuNCs probe for the sensitive and accurate detection of fluoroquinolones (FQs). Al^3+^ ions induced DIT@AgAuNCs aggregation, enhancing fluorescence through the aggregation-induced emission (AIE) effect. In the presence of FQs, which act as strong chelators for Al^3+^, the fluorescence emission of DIT@AgAuNCs at 615 nm decreased due to the disaggregation process. Additionally, a new emission peak emerged at 465 nm, likely resulting from the formation of a coordinated chelate between FQs and Al^3+^, providing an additional peak for ratiometric measurement of FQ concentration.

## 2. Experimental

### 2.1. Materials and reagents

Ciprofloxacin (CIP, 97.8%), ofloxacin (OFL, 98%), enrofloxacin (ENR), lincomycin (LNC, 98.5%), ampicillin (AMP, 97.4%), azithromycin (AZITH, 98.7%), gentamycin (GENT, 98.3%), roxithromycin (ROXI, 99.3%), streptomycin (STREP, 98.1%), and penicillin (PEN, 97.5%) were obtained as a gift from NOCAR, Giza, Egypt. Glucose (GLU, 96.4%), histidine (HIS, 99.3%), glutathione (GSH, 96.4%), cysteine (CYS, 97.4%), dithioerythritol (DIT, 98.5%), ascorbic acid (AA, 97.4%), uric acid (UA, 98.9%), dopamine (DA, 98.5%), chloroauric acid (HAuCl_4_·3H_2_O), and silver nitrate (AgNO_3_) were procured from Sigma-Aldrich. Sodium hydroxide (NaOH), hydrochloric acid (HCl), trichloroacetic acid, aluminum chloride hexahydrate (AlCl_3_·6H_2_O), phosphoric acid, acetic acid, and boric acid were obtained from Merck.

### 2.2. Instruments and samples preparation

All details are included in ESI.†

### 2.3. Preparation of DIT@AgAuNCs

A total of 155 mg of DIT was dissolved in 10 mL of double-distilled water (DDW), followed by sequential addition of 2.0 mL of 7.5 mM HAuCl_4_·3H_2_O and 3.5 mL of 2.2 mM AgNO_3_. The mixture was sonicated at 25 °C for 10 minutes, then adjusted to pH 10 by dropwise addition of 0.35 M NaOH. It was heated at 37 °C for 10 hours, dialyzed against DDW (2 kDa membrane) for 48 hours, and finally freeze-dried.

### 2.4. Detection of FQs

A mixture of 340 μL of 0.6 mg mL^−1^ DIT@AgAuNCs, 60 μL of 70.0 mM Al^3+^, and 400 μL of Britton–Robinson buffer (pH 6.0) was prepared and left at 25 °C for 1 minute. Then, 200 μL of FQs at varying concentrations was added, followed by 1.5 minutes incubation. Fluorescence was measured with excitation at 330 nm.

### 2.5. HPLC/UV conditions

FQs (CIP, NOR, and ENR) in egg, urine, and milk samples were quantified using HPLC-UV detection at 280 nm on a Kromasil C18 column (250 × 4.6 mm). The mobile phase consisted of phosphate buffer (pH 3.2) and acetonitrile (75 : 25, v/v), with a flow rate of 1.2 mL min^−1^. The injection volume was 10 μL. Under these conditions, the retention times were approximately 4.55 min for CIP, 5.12 min for NOR, and 5.87 min for ENR. The resolution values (*R*_s_) between adjacent peaks were all greater than 2.0, indicating good baseline separation and reliability for simultaneous quantification.

### 2.6. Statistical treatment

Data are expressed as mean ± standard deviation (SD) from independent experiments. Statistical analysis was performed using Origin software, with a two-tailed *t*-test (95% confidence level) used to compare the fluorometric and HPLC-UV methods.

## 3. Results and discussions

### 3.1. Characterization

The optical properties of the as-prepared DIT@AgAuNCs fluorescent probe were analyzed using UV-Vis spectroscopy (Fig. S1A†) and spectrofluorometry (Fig. S1B†). The probe exhibited an absorption band at 212 nm, attributed to the σ–σ* transition.^18,19^ Additionally, the absence of absorption in the 350–700 nm indicates the lack of surface plasmon resonance, confirming the absence of large nanoparticles.^20,21^ Fluorescence measurements revealed excitation and emission wavelengths at 330 nm and 615 nm, respectively, which were utilized for the subsequent determination of FQs. Notably, varying the excitation wavelengths caused a red shift in the fluorescence emission, indicating an uneven distribution of functional groups on the surface of DIT@AgAuNCs, Fig. S1C†.^22,23^ The stability of DIT@AgAuNCs was evaluated under various conditions, including pH (3.0–7.0), irradiation time (0–120 minutes), temperature (25–50 °C), and salt concentration (0.1–100 mM) (Fig. S2†). The results demonstrated that the fluorescent probe maintains a high degree of stability across these conditions, confirming its robustness for analytical applications. The quantum yield of the DIT@AgAuNCs and DIT@AgAuNCs/Al^3+^ were calculated to be 3.28% and 12.89%, respectively.

Fourier Transform Infrared (FTIR) spectroscopy was employed to confirm the functionalization of AgAuNCs with DIT (Fig. S3†). The FTIR spectrum of DIT exhibited characteristic absorption bands at 3365 cm^−1^, 2805–2910 cm^−1^, 2220 cm^−1^, and 1620 cm^−1^, corresponding to ν(OH), ν(CH_2_), ν(SH), and δ(OH) vibrations, respectively.^24,25^ Upon interaction with AgAuNCs, these absorption bands exhibited a blue shift, indicating a chemical interaction between the two components.^26^ Additionally, the disappearance of the absorption band at 2220 cm^−1^ confirms the formation of an Au–S covalent bond between DIT and AgAuNCs.

**Fig. 1:**
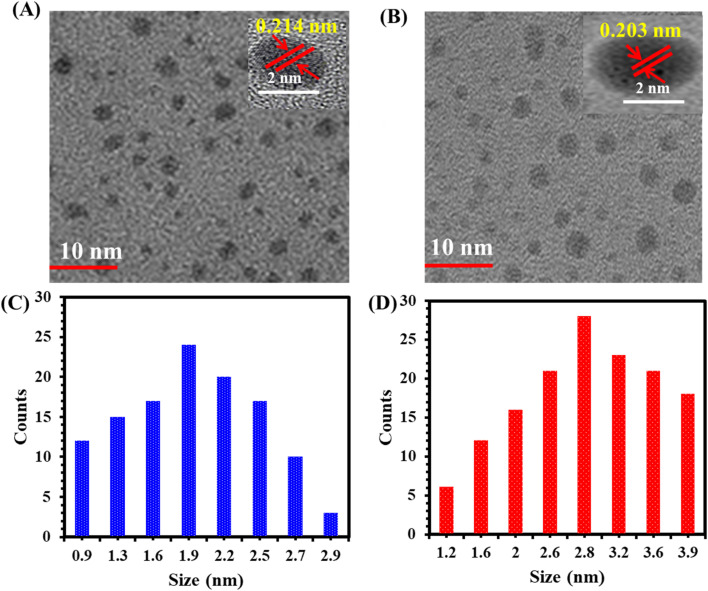
TEM images of (A) DIT@AuNCs and (B) DIT@AgAuNCs. (C) and (D) Depict the size distribution of DIT@AuNCs and DIT@AgAuNCs, respectively.

Fig. 1A presents the TEM image of DIT@AuNCs, revealing a spherical and well-dispersed morphology. The particle sizes range from 0.9 to 2.9 nm, with an average size of 1.90 nm (Fig. 1B). Upon Ag doping, the particle size slightly increased to 1.2–3.9 nm, with an average size of 2.80 nm (Fig. 1C and D). Furthermore, high-resolution TEM (HR-TEM) images of DIT@AuNCs and DIT@AgAuNCs demonstrate lattice spacings of 0.214 nm and 0.203 nm, respectively.^27,28^ Fig. S4A† illustrates the dynamic light scattering (DLS) analysis of DIT@AuNCs and DIT@AgAuNCs, revealing particle sizes of 17.89 nm and 118.2 nm, respectively. This significant increase in diameter confirms that Ag incorporation leads to the expansion of DIT@AgAuNCs compared to DIT@AuNCs. It is important to note that the particle sizes measured by DLS are larger than those observed in TEM due to the swelling effect of the particles in an aqueous environment.^29,30^ Fig. S4B† presents the energy-dispersive X-ray (EDX) spectrum of DIT@AgAuNCs, highlighting prominent peaks corresponding to carbon (C), oxygen (O), sulfur (S), gold (Au), and silver (Ag).

**Fig. 2:**
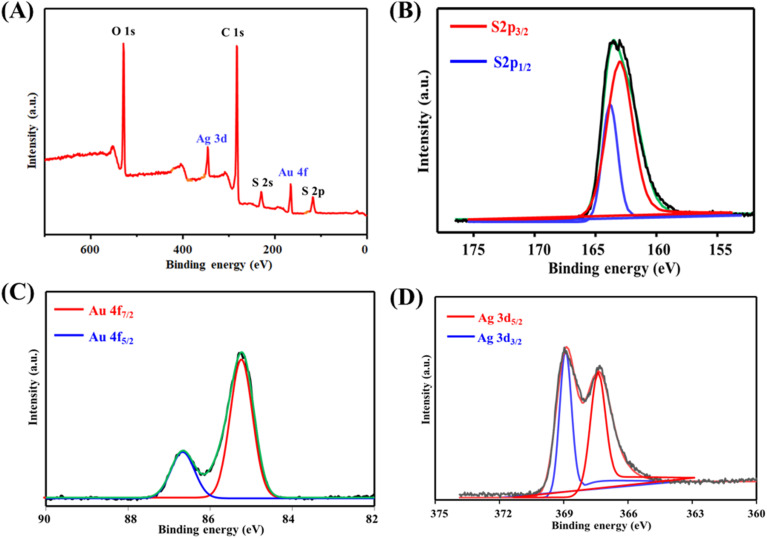
The full XPS spectrum of DIT@AgAuNCs (A). Panels (B–D) display the high-resolution spectra of S 2p, Au 4f, and Ag 3d, respectively.

The elemental composition and surface binding energies of DIT@AgAuNCs were analyzed using XPS, as presented in Fig. 2. The full survey spectrum (Fig. 2A) confirms the presence of C 1s, O 1s, S 2p, Au 4f, and Ag 3d, indicating the successful synthesis of the nanocomposite. The high-resolution S 2p spectrum (Fig. 5B) exhibits two distinct peaks at 163.7 eV and 164.7 eV, corresponding to S 2p_3/2_ and S 2p_1/2_, respectively. The deconvoluted Au 4f spectrum (Fig. 2C) reveals two characteristic peaks at 85.3 eV and 86.8 eV, assigned to Au 4f_7/2_ and Au 4f_5/2_, respectively. Similarly, the high-resolution Ag 3d spectrum (Fig. 2D) displays two well-defined peaks at 367.2 eV and 369.8 eV, attributed to Ag 3d_5/2_ and Ag 3d_3/2_, respectively. These spectral features confirm the successful integration of Ag and Au within the nanocomposite, further validating the structural and electronic characteristics of DIT@AgAuNCs.

### 3.2. Sensing mechanism

The addition of metal ions, such as Al^3+^, to DIT@AgAuNCs induces an aggregation-induced emission (AIE) effect, enhancing the fluorescence emission of these nanoclusters. This phenomenon can be attributed to the coordination interaction between Al^3+^ ions and DIT molecules, which restricts molecular vibrations and inhibits the free rotation of DIT. Consequently, this suppression of non-radiative relaxation pathways leads to an increase in fluorescence efficiency.^31,32^ To validate these findings, a series of morphological and spectroscopic investigations were conducted (Fig. S5†). The TEM image of DIT@AgAuNCs (Fig. S5A†) revealed an average diameter of 2.80 nm. Upon the incorporation of Al^3+^ ions, the TEM image (Fig. S5B†) showed a significant increase in particle size to approximately 27.89 nm, along with a well-defined aggregation pattern. The addition of CIP led to the disaggregation of these nanoclusters, as illustrated in Fig. S5C.† Moreover, DLS measurements were performed to determine the hydrodynamic diameters of DIT@AgAuNCs, DIT@AgAuNCs/Al^3+^, and DIT@AgAuNCs/Al^3+^/CIP, which were found to be 118.2 nm, 432.2 nm, and 152.89 nm, respectively (Fig. S6A†). The fluorescence lifetimes of DIT@AgAuNCs and DIT@AgAuNCs/Al^3+^ were measured to be 11.03 ns and 17.89 ns, respectively. This increase in fluorescence lifetime upon Al^3+^ binding provides strong evidence of AIE in the nanoclusters, as depicted in Fig. S6B.† These results confirm that the introduction of Al^3+^ ions induced the aggregation of DIT@AgAuNCs, while addition of CIP led to the disaggregation of the nanoclusters. Fig. S5D† presents the fluorescence spectra recorded for DIT@AgAuNCs, DIT@AgAuNCs/Al^3+^, CIP/Al^3+^, and DIT@AgAuNCs/Al^3+^/CIP. The weak fluorescence emission of DIT@AgAuNCs at 615 nm was significantly enhanced upon the addition of Al^3+^ ions due to the AIE effect. Additionally, the fluorescence emission peak of the Al^3+^-CIP coordinated complex appeared at 465 nm. Notably, the fluorescence spectrum of DIT@AgAuNCs/Al^3+^/CIP exhibited two distinct emission peaks at 615 nm and 465 nm, indicating the coexistence of both emission contributions.

**Fig. 3:**
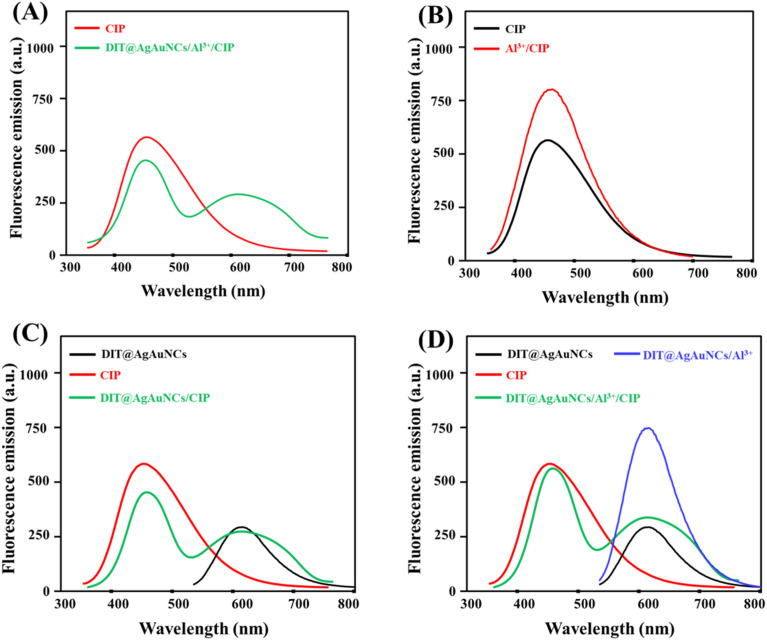
Fluorescence spectra of (A) CIP and DIT@AgAuNCs/Al^3+^/CIP, (B) CIP and Al^3+^/CIP, (C) DIT@AgAuNCs, CIP, and DIT@AgAuNCs/CIP, (D) DIT@AgAuNCs, DIT@AgAuNCs/Al^3+^, CIP, and DIT@AgAuNCs/Al^3+^/CIP.

It is essential to highlight the role of Al^3+^ ions and DIT@AgAuNCs in the detection of CIP, as well as how the fluorescence emission bands change in each system (Fig. 3). Previous studies have reported that trivalent metals, such as Al^3+^, can chemically interact with CIP through its carboxyl and pyridone groups.^33–35^Fig. 3A illustrates the fluorescence emission profiles of CIP and the DIT@AgAuNCs/Al^3+^/CIP system. It was observed that the emission of DIT@AgAuNCs at 615 nm decreased due to the coordination interaction between CIP and Al^3+^, while the emission band at 465 nm corresponds to the native fluorescence of CIP. Fig. 3B illustrates the role of Al^3+^ in enhancing the fluorescence emission of CIP. By binding to CIP, Al^3+^ inhibits excited-state intermolecular proton transfer (EISPT), thereby improving the fluorescence emission of CIP.^36^ The addition of CIP to DIT@AgAuNCs resulted in a reduction of CIP fluorescence emission, which may be attributed to Förster resonance energy transfer (FRET) between them, as illustrated in Fig. 3C. The presence of Al^3+^ ions in the system significantly increased the fluorescence intensity of both DIT@AgAuNCs and CIP (Fig. 3D). The detection process of the probe for CIP relies on its interaction with Al^3+^ ions, which facilitates CIP coordination. This interaction leads to the breakdown of DIT@AgAuNCs aggregates, causing a reduction in their red fluorescence while maintaining the inherent blue emission of CIP.

### 3.3. Optimization of variables

To enhance the sensitivity of the fluorescent probe, reaction conditions for the interactions between DIT@AgAuNCs and Al^3+^, as well as DIT@AgAuNCs/Al^3+^ and FQs, were optimized. Fig. S7† illustrates key influencing factors. Fig. S7A† shows that the interaction was optimal at pH 6.0, while DIT@AgAuNCs emission remained stable across pH 4.0–8.0. Fig. S7B† indicates that fluorescence emission peaked at an Al^3+^ concentration of 70.0 mM, which was used throughout the study. Fig. S7C† reveals that 1.0 minutes was the optimal reaction time, and Fig. S7D† confirms 25 °C as suitable for interaction.

Reaction conditions for the DIT@AgAuNCs/Al^3+^-FQs system were optimized, focusing on pH and reaction time. Fig. S8A† shows that maximum fluorescence occurred at pH 6.0. Acidic conditions weaken Al^3+^-FQ coordination due to the protonation of functional groups such as the carboxyl and keto groups on the FQ molecule, which diminishes their ability to chelate with Al^3+^ ions, consistent with previous findings.^33–35^ Fig. S8B† indicates that fluorescence response peaked at 1.5 minutes, confirming it as the optimal reaction time.

### 3.4. Detection of FQs

**Fig. 4:**
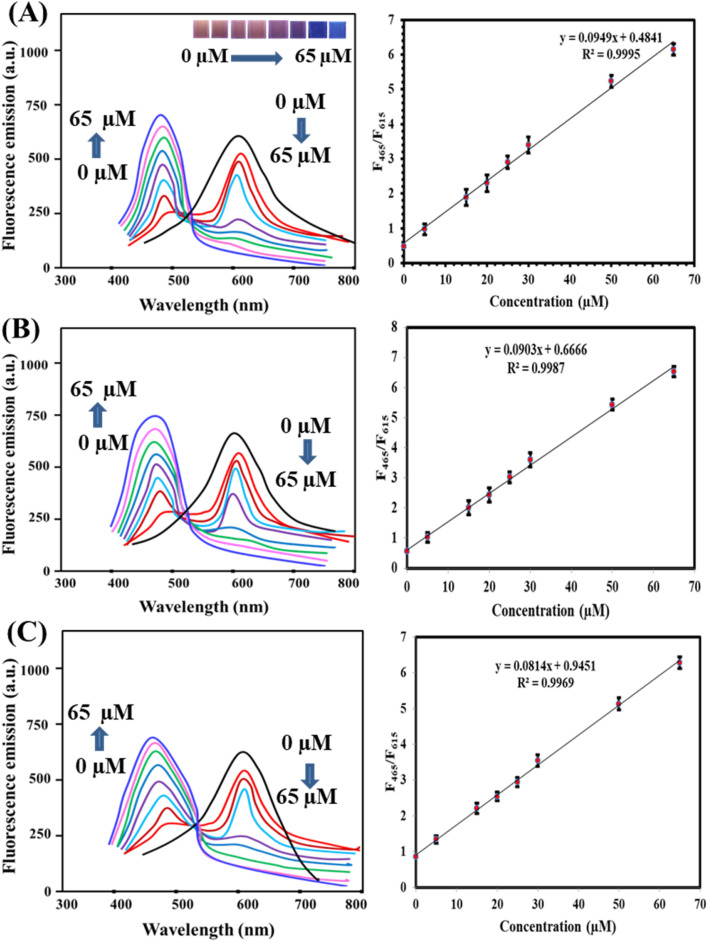
Fluorescence spectra of (A) CIP, (B) NOR, and (C) NOR along with their calibration plots showing a relationship between (F_465_/F_615_) and FQs' concentration. The number of replicates was five.

The fluorescence sensing interface was used to detect FQs, including ciprofloxacin (CIP), norfloxacin (NOR), and enrofloxacin (ENR). Under optimal conditions, the emission intensity of DIT@AgAuNCs/Al^3+^ at 615 nm decreased with increasing FQ concentrations, while the 465 nm emission band intensified due to FQs-Al^3+^ complex formation. Fig. 4A–C presents the fluorescence spectra of CIP, NOR, and ENR at 615 nm and 465 nm, showing a concentration-dependent increase in the fluorescence responses (*F*_465_/*F*_615_) within the ranges of 0.01–60 μM, 0.018–60 μM, and 0.021–60 μM, respectively. The detection limits, based S/N ratio of 3 : 1, were determined as 3.1 nM for CIP, 3.8 nM for NOR, and 4.4 nM for ENR. To demonstrate the analytical superiority of the proposed sensor, Table 1 compares its performance with previously reported probes. For instance, Fu *et al.*^1^ shows strong conceptual similarity to our study, as both employ Al^3+^-mediated modulation of nanocluster fluorescence and utilize a ratiometric sensing mechanism for CIP detection. However, our probe distinguishes itself through several enhancements. Firstly, we introduce a bimetallic AgAu NCs system, which demonstrates improved quantum yield, enhanced AIE effects, and greater stability compared to the monometallic gold clusters used in ref. 1. Secondly, while Fu *et al.*'s probe targets CIP exclusively, our system effectively detects multiple FQs (CIP, NOR, ENR) with comparable sensitivity and rapid response. Additionally, our probe preparation is straightforward and does not require post-synthetic surface modification or ligand exchange. These distinctions position our system as a broader and more practical platform for monitoring FQs residues across diverse matrices, including food and biological samples. The copper nanocluster probe by Hosseini and Sadeghi^15^ achieved a detection limit of 9.0 nM for CIP, which is less sensitive than the current sensor. Likewise, the riboflavin/CD-based sensor developed by Lu *et al.*^36^ reported a detection limit of 130 nM, substantially higher than our proposed method (3.1 nM). These comparisons highlight the enhanced sensitivity of our platform. The DTE-Cu NCs/Tb^3+^ probe^40^ offers ratiometric detection but has a narrower linear range (0.032–20 μM) compared to our method. Similarly, probes such as Eu/GMP NPs^41^ and Cys-CuNCs^42^ demonstrate ratiometric capabilities but require longer response times. In contrast, the current DIT@AgAuNCs/Al^3+^ probe benefits from a simple design and rapid reaction kinetics based on aggregation/disaggregation mechanisms mediated by Al^3+^ and FQ coordination. Unlike systems that involve complex synthesis (*e.g.*, MIP-QDs) or rely on single-emission strategies prone to background interference, our probe achieves lower detection limits, faster analysis, and improved accuracy through a ratiometric readout. These advantages make it well-suited for real-time applications in food safety and environmental monitoring.

**Table 1:** Comparison between performances of DIT@AgAuNCs/Al^3+^ and other reported fluorescence probes used for determination of FQs^a^

Probe	FQs	Mode	Response time (minutes)	Linear range (μM)	LOD (μM)	Reference
AuNCs/Al^3+^	CIP	Turn-on, ratiometric	1	0.01–12	0.0014	1
Gl@CuNCs	CIP	Turn-on, single	5	0.015–0.9	0.009	15
Riboflavine/CDs	CIP	Turn-on, ratiometric	1	0.5–200	0.13	36
SQDs	CIP	Turn-on, single	1	0.02–1.0	0.005	37
	NOR	Turn-on, single	1	0.02–1.25	0.007	
CDs	NOR	Turn-on, ratiometric	3	1–70	0.007	38
MIP-QDs	NOR	Turn-off	3	0.5–28	0.18	39
DTE-Cu NCs/Tb^3+^	NOR	Turn-on, ratiometric	0.5	0.032–20	9.6	40
	ENR	Turn-on, ratiometric	0.5	0.025–22.5	7.7	
Eu/GMP NPs	CIP	Turn-on, single	15	1–40	0.78	41
Cys-CuNCs	NOR	Turn-on, single	20	0.5–50	0.05	42
DIT@AgAuNCs/Al^3+^	CIP	Turn-on, ratiometric	1.5	0.01–60	0.0031	This work
	NOR	Turn-on, ratiometric	1.5	0.018–60	0.0038	
	ENR	Turn-on, ratiometric	1.5	0.021–60	0.0044	

a CDs: carbon dost; SQDs: sulfur quantum dots; MIP: molecularly-imprinted polymers; QDs: quantum dots; DTE: dithioerythritol; NCs: nanoclusters; GMP NPs: guanosine monophosphate nanoparticles; CQDs: carbon quantum dots; RhB: rhodmaine B.

### 3.5. Selectivity

**Fig. 5:**
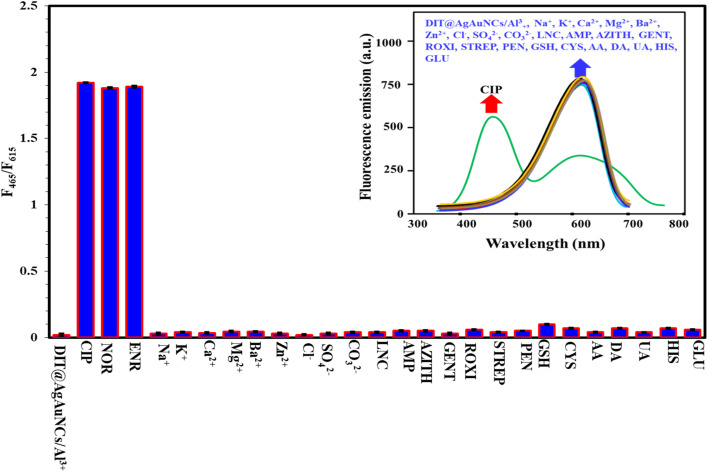
Fluorescence responses of DIT@AgAuNCs/Al^3+^-based probe for detection of 15 μM FQs and ten folds of other interfering species (common ions, small biomolecules, and other antibiotics). The number of replicates was five.

Selectivity is a crucial factor in assessing a probe's detection performance. Herein, the ratiometric fluorescence response of the DIT@AgAuNCs/Al^3+^-based probe was tested against various potential interfering substances. As illustrated in Fig. 5, only FQs (CIP, NOR, ENR) significantly altered the fluorescence intensity ratio (*I*_465_/*I*_615_). Other potential interfering species, including common ions (Na^+^, K^+^, Ca^2+^, Mg^2+^, Ba^2+^, Zn^2+^, Cl^−^, SO_4_^2−^, CO_3_^2−^), small biological molecules (GSH, CYS, AA, DA, UA, HIS, GLU), and antibiotics (LNC, AMP, AZITH, GENT, ROXI, STREP, PEN) had minimal influence on the probe's fluorescence intensity ratio, even at concentrations ten times higher than those of FQs. These findings proved that the proposed probe has good selectivity towards FQs detection.

### 3.6. Applications

The feasibility of the DIT@AgAuNCs/Al^3+^ fluorescent probe for FQ detection *e.g.* CIP was validated using egg, milk, and urine samples. Protein interference in egg and milk was removed with trichloroacetic acid. The probe demonstrated high accuracy, with recoveries of 94.0–106.0%, and reliability, with an RSD below 4.09% (Table 2).

**Table 2:** Application of DIT@AgAuNCs/Al^3+^ and HPLC/UV methods for CIP detection in egg, milk, and urine samples (*n* = 5)

Sample	Added (μM)	DIT@AgAuNCs/Al^3+^			HPLC/UV			*t*-Calculated^a^
		Found (μM)	Recovery (%)	RSD (%)	Found (μM)	Recovery (%)	RSD (%)	
**Egg**								
Sample 1	0.5	0.51	102.0	2.67	0.53	106.0	3.45	1.673
	1.5	1.47	98.0	3.67	1.55	103.3	3.54	1.762
	2.0	1.98	99.0	2.78	1.92	96.0	4.23	2.093
Sample 2	0.5	0.52	104.0	2.04	0.47	94.0	3.56	1.537
	1.5	1.53	102.0	2.78	1.54	102.7	2.98	1.783
	2.0	2.07	103.5	3.65	2.07	103.5	3.76	1.980

**Milk**								
Sample 1	1.5	1.59	98.0	3.67	0.47	94.0	2.70	1.864
	2.0	2.09	106.0	3.55	1.56	104.0	3.45	1.934
	0.5	0.47	104.5	2.78	1.93	96.5	4.76	2.094
Sample 2	1.5	1.48	94.0	2.89	0.52	104.0	3.29	1.840
	2.0	2.05	98.7	3.21	1.56	104.0	4.35	1.934
	1.5	1.59	102.5	4.09	2.05	102.5	3.87	1.993

**Urine**								
Sample 1	0.5	0.52	104.0	3.29	0.52	104.0	2.76	1.453
	1.5	1.53	102.0	2.54	1.56	104.0	3.49	1.730
	2.0	2.04	102.0	3.27	1.95	97.5	4.29	1.983
Sample 2	0.5	0.51	102.0	3.27	0.51	102.0	3.28	1.530
	1.5	1.56	104.0	3.29	1.54	102.7	3.87	1.762
	2.0	2.07	103.5	3.20	2.05	102.5	4.36	1.982

a Indicates a statistically significant difference at *P* < 0.05 (*t*-calculated > *t*-tabulated = 2.132).

## 4. Conclusion(s)

This study introduces a ratiometric fluorescent probe for FQ detection, utilizing the aggregation-induced emission (AIE) properties of DIT@AgAuNCs triggered by Al^3+^ ions. The coordination of Al^3+^ with hydroxyl and thiol groups on DIT@AgAuNCs enhanced fluorescence, while FQs binding to Al^3+^*via* carboxyl and carbonyl groups activated their intrinsic fluorescence. Under optimal conditions, the probe enabled a rapid (1.5 min) and sensitive response to CIP, NOR, and ENR, demonstrating high recovery and stability in detecting FQ residues in eggs, milk, and urine. This work provides a reliable strategy for monitoring FQs in animal-derived foods and a foundation for developing rapid food safety analysis platforms. However, limitations include the inability to distinguish between different FQs in complex mixtures and the lack of testing in more challenging biological matrices like blood. Future work should focus on enhancing probe selectivity and specificity through the integration of molecular recognition elements, expanding the detectable antibiotic spectrum, and developing portable, field-deployable platforms. These efforts will further improve the practical application of this sensing strategy for food safety, environmental monitoring, and clinical diagnostics.

## Conflicts of interest

The authors declare no competing interests.

## Supplementary Material

RA-015-D5RA02878G-s001

## Data Availability

Data will be available upon request from the corresponding authors.
