# Supplementary material for: Dithioerythritol-capped silver/gold nanoclusters for determination of ciprofloxacin, norfloxacin, and enrofloxacin in food and urine samples
Source: RSC Adv. 2025 Jul 7;15(29):23364–73. doi: 10.1039/d5ra02878g (PMC12230799; doi:10.1039/d5ra02878g)
Supplement: RA-015-D5RA02878G-s001 [file RA-015-D5RA02878G-s001.pdf]

Supplementary Information (SI) for RSC Advances.
This journal is © The Royal Society of Chemistry 2025

Electronic Supplementary Material

Dithioerythritol-capped silver/gold nanoclusters for determination of ciprofloxacin, norfloxacin, and enrofloxacin in food and urine samples

Mohamed N. Goda[a], Laila S. Alqarni[a], Hossieny Ibrahim[b] , Al-Montaser Bellah H. Ali [c], Mohamed M. El-Wekil [c*]

[a] Department of Chemistry, College of Science, Imam Mohammad Ibn Saud Islamic University (IMSIU), Riyadh 11623, Saudi Arabia

[b] Department of Chemistry, Faculty of Science, Assiut University, Assiut 71516, Egypt

[c] Department of Pharmaceutical Analytical Chemistry, Faculty of Pharmacy, Assiut University, Assiut 71516, Egypt

Correspondence

mohamed.elwakeel@pharm.aun.edu.eg, mohamed.mohamoud@ymail.com

Instruments and samples preparation

The morphology of the DIT@AgAuNCs was investigated using TEM (Transmission Electron Microscope, Jeol F200, Japan). Nicolet 6700 (USA) Fourier transform infrared spectroscopy was used to reveal the surface functional groups of DIT@AgAuNCs. The elemental composition and binding state were demonstrated using X-ray photoelectron spectroscopy (thermo Scientific, USA). Dynamic light scattering (DLS) and zeta potential were measured using ZetaSizer (Malvern, USA). Fluorometric and spectrophotometric measurements were conducted using RF-5301 (slit width= 5 nm) and spectrophotometer, respectively (Shimadzu Ltd., Tokyo, Japan).

Preparation of samples

The proposed fluorescence probe was utilized to detect FQs in real samples, including egg, milk, and urine. The probe was applied both before and after spiking with varying concentrations of a standard drug using the spiking technique. For the egg sample, 0.75 g of egg was mixed with different amounts of FQs. Then, 3.5 mL of trichloroacetic acid was added, followed by centrifugation at 6000 rpm for 20 minutes. Finally, the collected supernatant was analyzed using the proposed fluorescence sensor (Section 2.4).

For milk analysis, 300 μL of milk was mixed with varying concentrations of FQs, followed by the addition of 5.5 mL of trichloroacetic acid and centrifugation at 8000 rpm for 15 minutes. The supernatant was then analyzed using the fluorescence sensor (Section 2.4).

For urine analysis, 200 μL of human urine was mixed with varying concentrations of FQs, centrifuged at 4000 rpm for 10 minutes, and the resulting supernatant was similarly analyzed using the fluorescence sensor (Section 2.4).

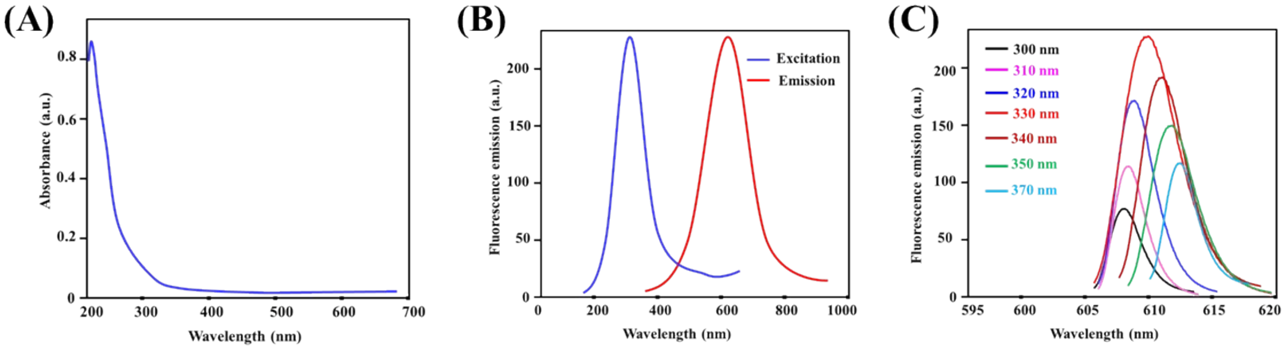

Fig.S1 presents the optical characteristics of DIT@AgAuNCs, including (A) the absorption spectrum and (B) the fluorescence spectrum. (C) Illustrates the emission dependency on excitation wavelengths, providing insight into the excitation-dependent photoluminescence behavior of the DIT@AgAuNCs.

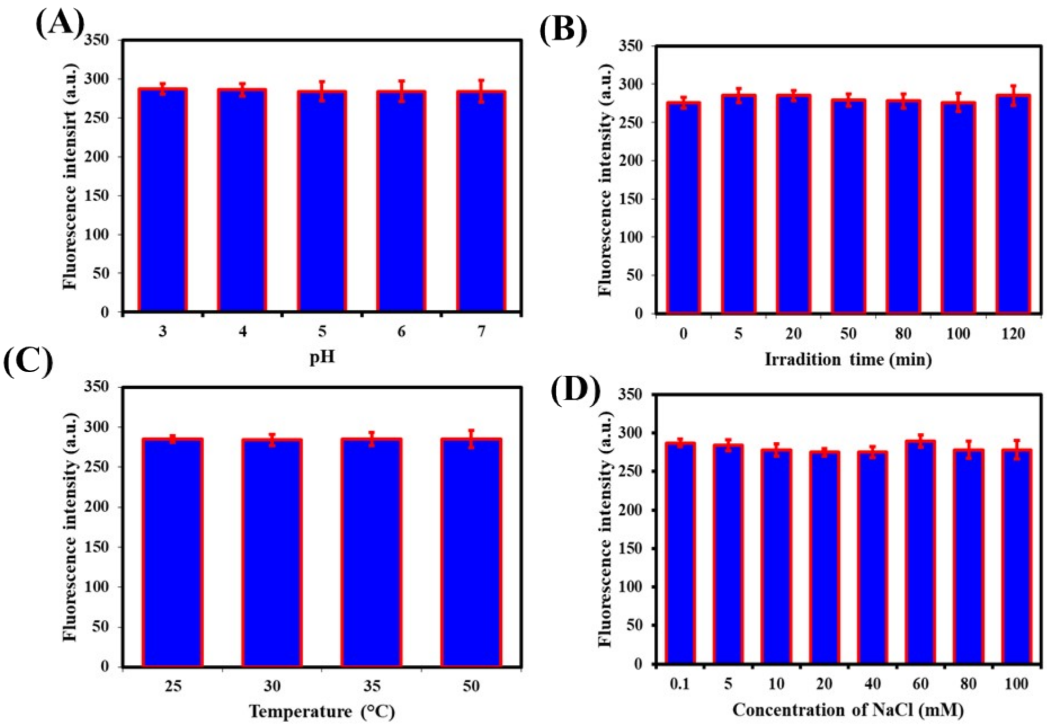

Fig.S2 illustrates the factors influencing the stability of DIT@AgAuNCs, including the effects of (A) pH, (B) irradiation time, (C) temperature, and (D) NaCl concentration. The number of replicates was five.

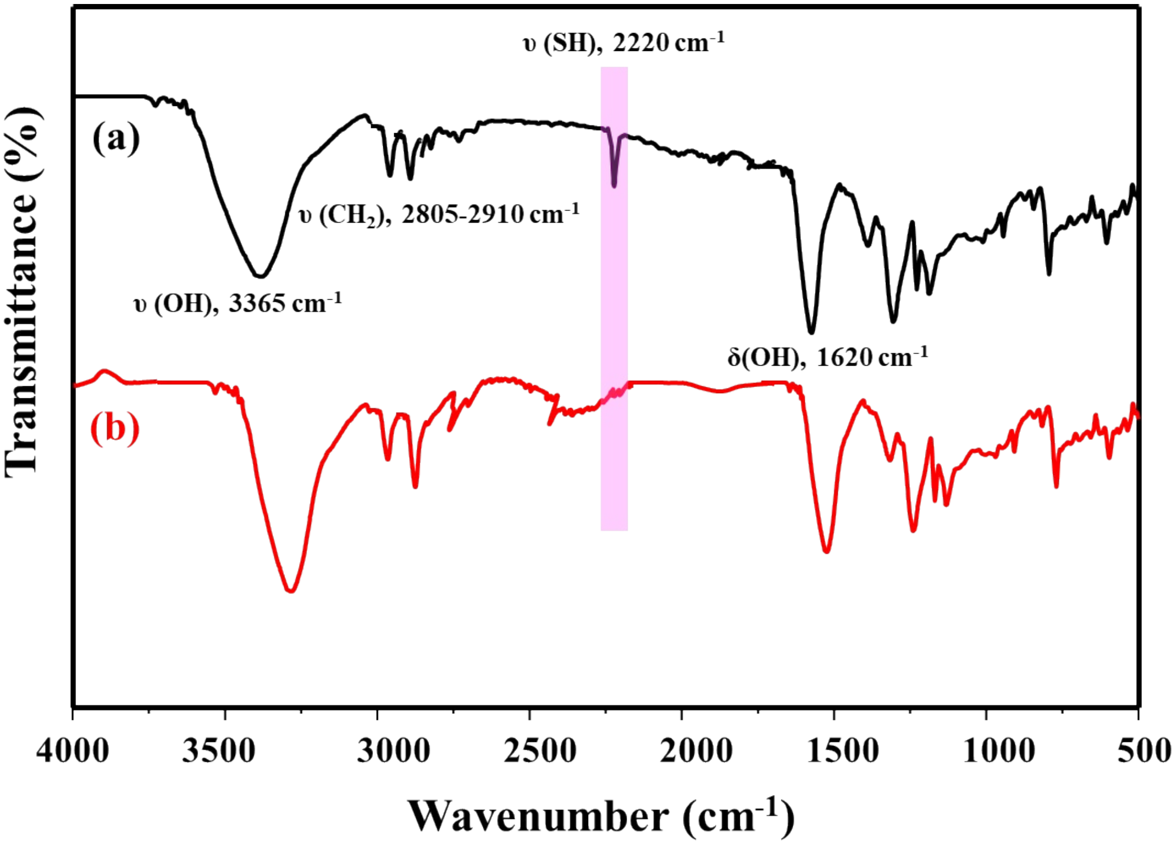

Fig.S3 The FTIR spectra of (a) DIT and (b) DIT@AgAuNCs.

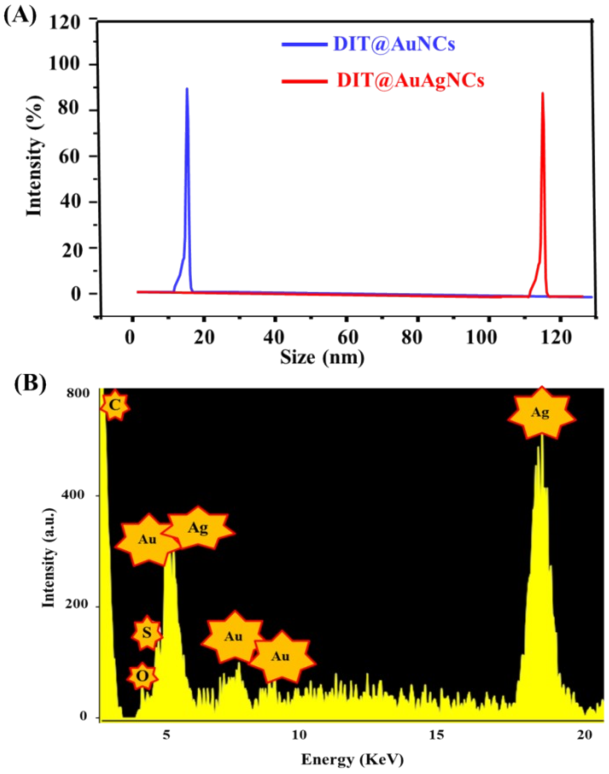

Fig.S4 (A) DLS of DIT@AuNCs and DIT@AgAuNCs. (B) EDX of DIT@AgAuNCs.

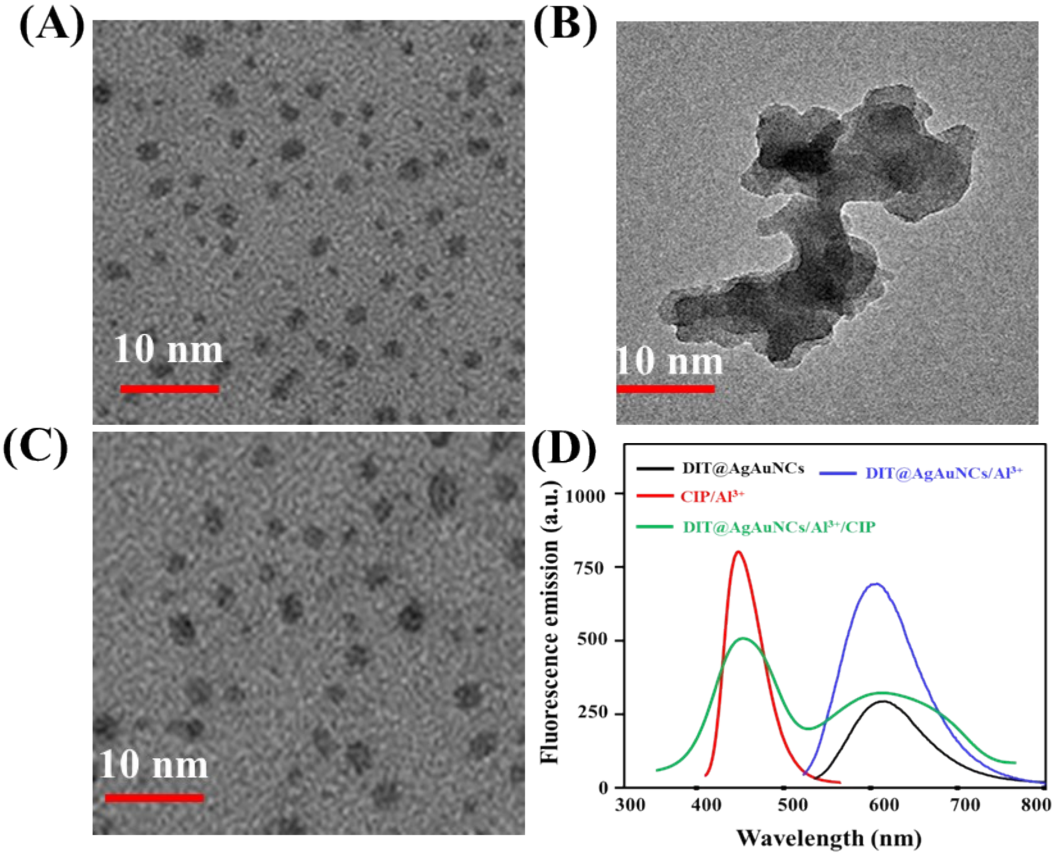

Fig.S5 TEM images of DIT@AgAuNCs (A), DIT@AgAuNCs/Al^{3+} (B), and DIT@AgAuNCs/Al^{3+}/CIP (C) while (D) is fluorescence spectra of DIT@AgAuNCs, DIT@AgAuNCs/Al^{3+}, CIP/Al^{3+}, and DIT@AgAuNCs/Al^{3+}/CIP.

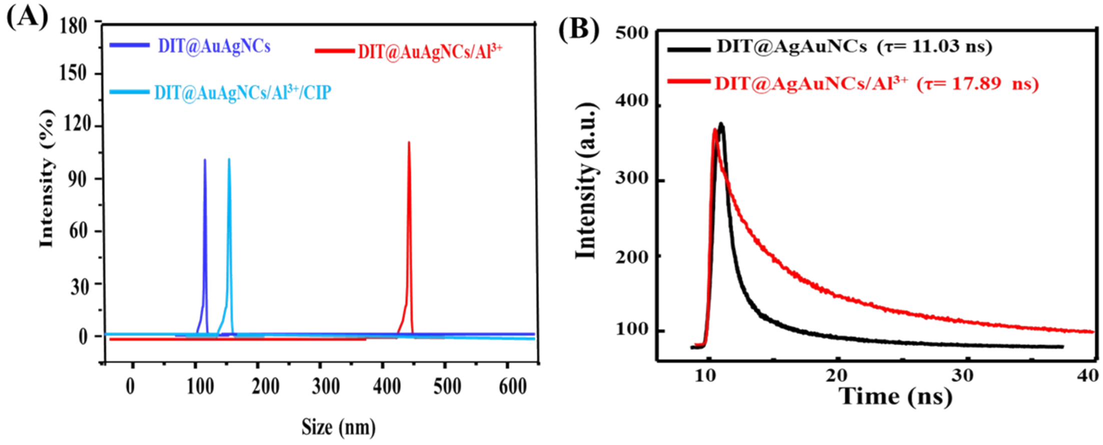

Fig.S6 (A) DLS of DIT@AgAuNCs, DIT@AgAuNCs/Al^{3+}, and DIT@AgAuNCs/Al^{3+}/CIP. (B) Fluorescence lifetimes of DIT@AgAuNCs before and after addition of Al^{3+}.

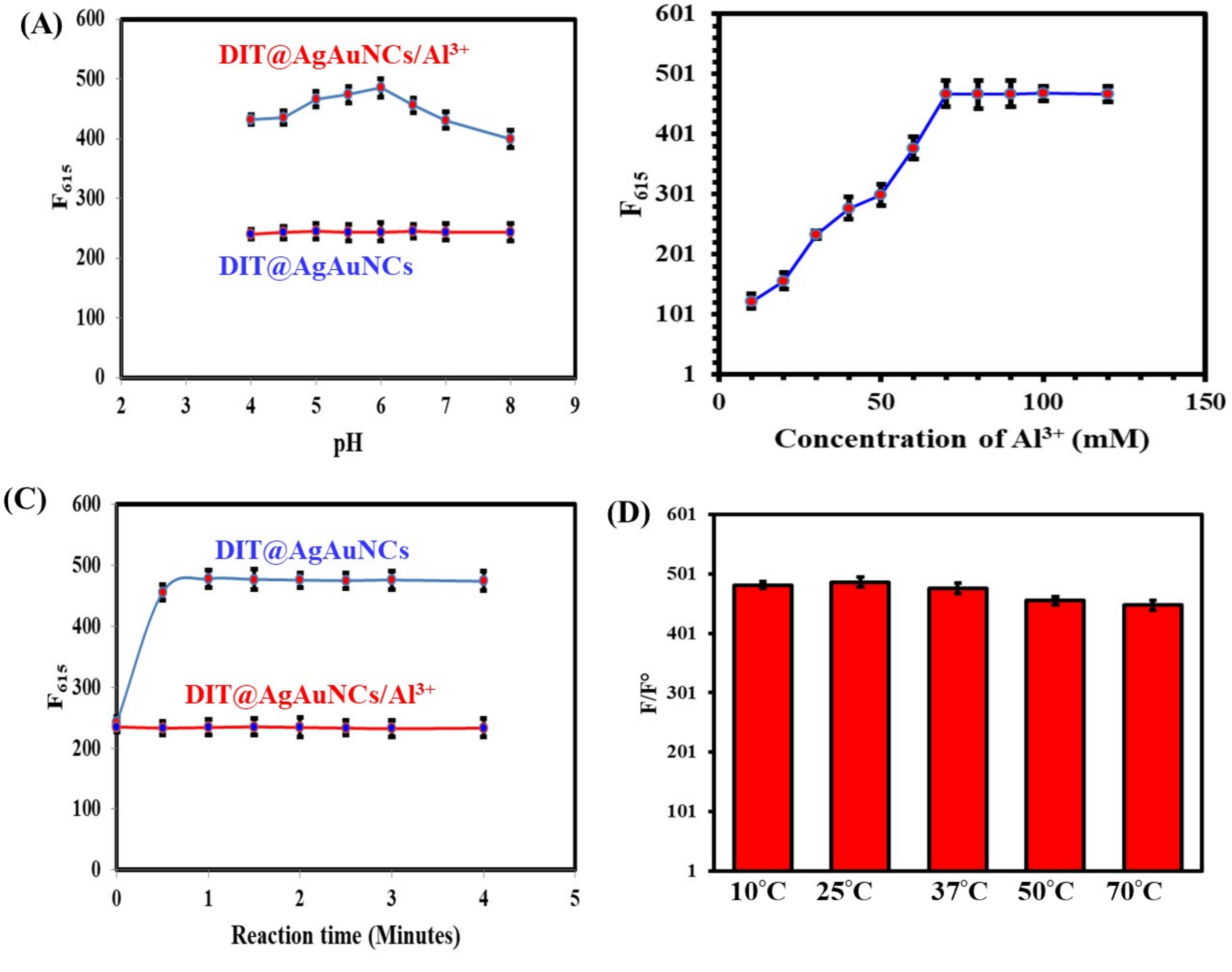

Fig.S7 The effect of pH (A), Al^{3+} ion concentration (B), reaction time (C), and reaction temperature (D) on the fluorescence emission of DIT@AgAuNCs induced by Al^{3+} ion. The number of replicates was five. F/F^0 denotes the relative fluorescence intensity at a specific wavelength at specific temperature degree (F) compared to the blank or baseline measurement (F_o).

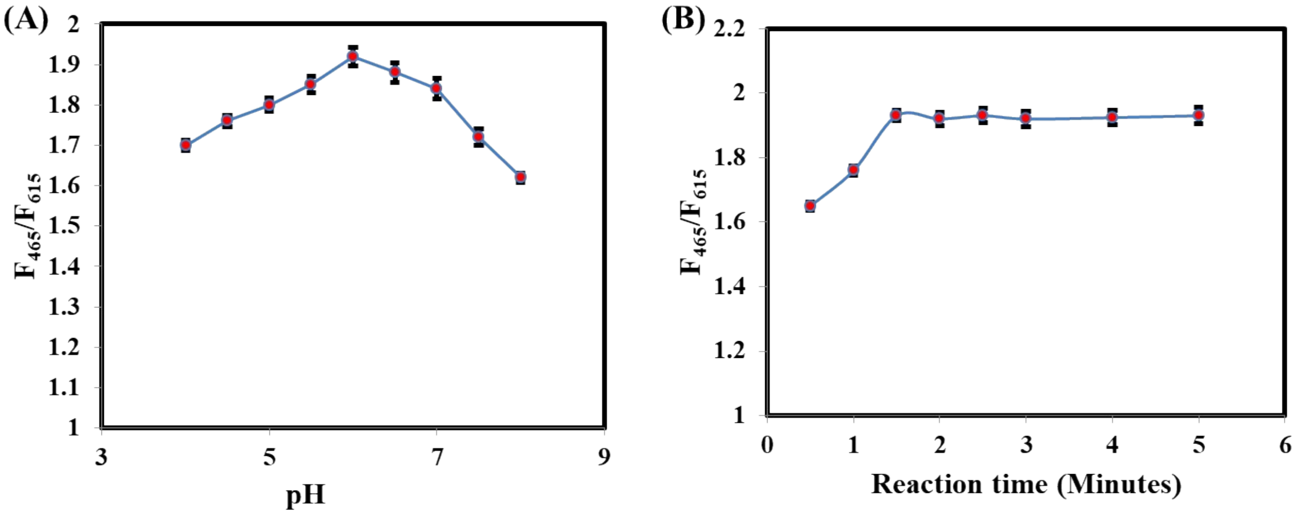

Fig. S8 The effect of pH (A) and reaction time (B) on the fluorescence response of by DIT@AgAuNCs/Al^{3+} induced by 15.0 μM CIP. The number of replicates was five.
